# Alterations to proteins in the lens of hereditary *Crygs*-mutated cataractous mice

**Published:** 2010-06-11

**Authors:** Yinghong Ji, Hua Bi, Na Li, Hong Jin, Pengyuan Yang, Xiangyin Kong, Shunsheng Yan, Yi Lu

**Affiliations:** 1 Department of Ophthalmology, Eye & ENT Hospital, Fudan University, Shanghai, PR China; 2 College of Optometry, Nova Southeastern University, Fort Lauderdale, FL; 3 Department of Chemistry, Fudan University, Shanghai, PR China; 4 Institute of Health Sciences, Shanghai Institutes for Biological Sciences, Chinese Academy of Science, Shanghai, PR China; 5 Xinjiang Institute for Endemic Control and Research, Wurumuqi, Xinjiang, PR China

## Abstract

**Purpose:**

To investigate the altered expression of proteins in the lens of mice with inherited cataracts.

**Methods:**

Mice with inherited cataracts  caused by a spontaneous mutation of the gene gamma S-crystallin (*Crygs*)** were used as the subjects. Lens proteins were extracted and separated by two-dimensional electrophoresis (2-DE). The spots representing differential proteins were first identified by image analysis, and then further analyzed by matrix assisted laser desorption/ionization-time of flight-tandem mass spectrometry (MALDI-TOF-MS/MS).

**Results:**

2-DE were conducted under high (882 μg) and low dosage (190 μg) of sample. Under each condition, the numbers of protein spots found in cataract lenses were similar to those in normal lenses (p>0.05). Seventeen proteins were identified in normal lenses, including αA- to αB-, βA1- to βA4-, βB1- to βB3-, γA- to γF-, and γS-crystallin, and bead-filament structure protein (BFSP/filensin). Seven differential ones were consistently identified. In the cataract lenses BFSP and γS-crystallin were absent; γF-crystallin was downregulated; and βA1-, βB1-, βB2-, and αB-crystallin were upregulated. Those abnormally upregulated crystallins, when compared to normal ones, had smaller molecular weight, suggesting possible truncation.

**Conclusions:**

The mutant *Crygs* gene can lead to changes of BFSP/filensin and other crystallins. The changes to these crystallins, together, may secondarily lead to cataract formation.

## Introduction

Studying the composition and sequences of proteins of lenses, both normal and cataractous, is critical to understanding the formation of cataracts. Ideally, experiments should be performed directly on lenses collected from patients with various forms of cataracts. However, due to certain clinical concerns, it is rather difficult to do so. Therefore, most of the current studies incorporating proteomic analysis on lenses have been done in rodents. Lampi [1] and Ueda [2] provide two-dimensional electrophoresis (2-DE) maps of normal lens tissues from rats and mice. There are also studies about the proteomic analysis of lens epithelium [3,4]. For studies on cataractous lenses, previous research has been conducted on rodent cataractous models formed by transgenic manipulations,  gene knockout or spontaneous mutation. Tumminia [5] has investigated the difference of 2-DE images between transgenic mouse TG72 containing the HIV-1 protease linked to the lens αA-crystallin promoter and normal mice. Mou et al. [6] explored the target genes of heat shock transcription factor 4 (*HSF4*), especially those involved in lens developmental processes and cataract formation, using *Hsf4*-knockout mice. Hoehenwarter et al. [7] analyzed the urea soluble proteins of the lens of two mouse strains, C57BL/6J and 129/SvJ with the disruption of the alpha3Cx46 connexin gene (*Gja3*) by a comparative proteomics approach. Takeuchi [8] and Kamei [9] compared crystallin proteins of normal rats with that of cataract ICR/f mutant rats, using gel filtration and two-dimensional gel electrophoresis. They also measured the transglutaminase activities, Ca^2+^ content and the phosphorylation modification of alpha-crystallin in the mutant and normal lenses.

In this study, the subjects used were mice with a form of spontaneous congenital cataract. Mice with this unusual characteristic were first discovered incidentally by the Xinjiang Institute for Endemic Control and Research. After ten years of inbreeding, these mice were established as a recessive-type hereditary cataract strain. Through gene mapping, it was determined to be a gamma S-crystallin (*Crygs*) mutation by the Institute of Health Sciences, Shanghai Institutes for Biologic Science and the Chinese Academy of Science. Fifteen days after mice of that strain are born, bilateral cataracts can be clearly observed. By 35 days, the cataracts become mature. Since the unique characteristics of spontaneous development and inheritance in this mouse model are very close to the way cataracts develop in humans, studying the compositions of proteins in these mouse lenses will greatly enhance our understanding of the formation of cataracts in humans. In this study, we applied comparative proteomic analysis, combined with 2-DE and mass spectrometry (MS) to study the difference in protein components between normal lenses and cataract lenses.

There are three major families of crystallins (α-, β-, and γ-crystallin) in the lenses of mammals. Gamma crystallins have seven members, γA- to γF- and γS-crystallin. Mice have all seven. Three of them, γC-, γD-, and γS-crystallin, constitute the bulk of the γ-crystallins in the human lens. The γ-crystallins, except γS-crystallin, are synthesized primarily in the early stages of development, so that the content of γ-crystallin is greater in the lens nucleus than in the younger cortical regions of the lens. The expression of γS-crystallin increases after birth and continues throughout life [10]. The mice used in this study had symmetric binocular nuclear cataracts. These are caused by a single point mutation, G489A, in *Crygs* [11]. Compared to normal mice, all other genes are normal. Therefore, this particular mouse is an ideal subject to study the role of γS-crystallins in cataract formation.

## Methods

Solutions and test kits: Immobilized pH gradient (IPG) gel strips (18 cm, pH 3–10, linear) and IPG buffer (pH 3–10, linear) were provided by Bio-Rad (Berkeley, CA). Urea, 3-[(3-cholamidopropyl)dimethylammonio]-1-propane sulphonate (CHAPS), dithiotheritol (DTT), thiourea, phenylmethyl sulfonyl fluoride (PMSF), bromphenol blue, mineral oil, glycine, glycerol, acrylamide, N,N'-methylenebisacrylamide, tetremethylenedianmine (TEMED), ammonium persulfate (APS), α-Cyano-4-hydroxycinnamic acid (CHCA), ammonium citrate, trifluoroacetic acid (TFA), and myoglobin were provided by Sigma (St. Louis, MO). Acetonitrile (ACN) and lodoacetamide were supplied by Fluka (St. Louis, MO). Ethanol, phosphoric acid, acetic acid, agarose, Coomassie brilliant blue (CBB) and other routine reagents were all bought from Sinopharm Chemical Reagent Co., Ltd (Shanghai, China). Bovine serum albumin (BSA) was provided by Shenneng Boxin Biotechnology Inc. (Shanghai, China). Trypsin, sequencing grade, was provided by Roche (Indianapolis, IN). Deionized water was produced using a Millipore system (Millipore, Bedford, MA).

### Equipment

IPGphor horizontal isoelectric focusing electrophoresis was purchased from Amersham Pharmacia (Piscataway, NJ). A Power/PAC 1000 vertical electrophoresis, GS-800 gel imaging system, and PDQUEST 7.30 2-DE image analysis software were provided by Bio-Rad. 4700 Proteomics Analyzer (TOF/TOF^TM^) was purchased from Applied Biosystems Inc. (Foster City, CA). The search engine was GPS (Applied Biosystems) – MASCOT (Matrix Science, London, UK). A high-speed low-temperature centrifuge was provided by Sigma. Deionized water was produced using a Millipore system (Millipore, Bedford, MA). The UV spectrophotometer (UV mini 1240), TS-1 decolorization shaker, and XW-80A vortex mixer were obtained from Kirin Medical Equipments (Haimen, Jiangsu, China).

### The extraction of crystalline lenses and proteins

Normal (male, n=3) and congenital inherited cataract Kunming mice (male, n=3) were provided by the Experimental Animal Center of the Chinese Academy of Science, Shanghai, China. Animal care and handling was provided in accordance with the Institutional Animal Care and Use Committee guidelines. These mice were SPF grade and aged 5 weeks. In normal mice, the crystalline lenses were transparent. However, in those mice with congenital inherited cataracts, lenses showed dense central nuclear cataracts. The mice were killed by spine dislocation. Eyeballs from both eyes were removed under Zeiss microscope and rinsed in Ringer’s solution. While the eyeballs were bathed in Ringer’s solution, an incision was made on the posterior part of the sclera and the crystalline lenses were removed intact. Each cataract or normal group had six crystalline lenses. The removal of the lens capsule was performed under Zeiss microscope. The cystitome, made by bending a 27 gauge insulin needle, with a very sharp cutting tip was brought to rest at the center of the capsule and used to make a small radial opening. The cystitome was inserted just below the capsule, and raised to create a small capsular flap. The flap, caught with forceps at one side and needle tip at the other side, was pulled carefully in opposite directions. Since the connections between the capsule and the rest of the lens were relatively loose, the capsule could be entirely peeled off without removing the sub-capsule cortex. Once the whole lens was exposed, it was immediately preserved in liquid nitrogen. The crystalline lenses were then crushed into powder and total proteins were extracted with 800 μl lysis buffer (7 M Urea, 4 % CHAPS, 2 M Thiourea, and 0.14 % PMSF) by homogenizing for 30 min on ice and centrifuged at 12,000× g and 15 °C for 30 min. The supernatant was liquated and stored at −80 °C before use. Protein concentrations of all the samples were determined by Bradford method. Briefly, prepare standard concentrations of BSA of 0.5 µg/µl and add a series of sample of 0, 5, 9, 13, 17, 21, 25, and 29 µl. Add 100 µl of each of the above to separate tubes and add 1.0 ml of Coomasie Blue to each tube. Turn on and adjust a spectrophotometer to a wavelength of 595 nm, and blank the spectrophotometer using 1.5 ml cuvettes. Wait 3 min and read the absorbance of each standard and sample at 595 nm. Plot the absorbance of the standards versus their concentration. Compute the extinction coefficient and calculate the concentrations of the unknown samples. 

### 2-DE and image analysis

Samples of 882 μg and 190 μg were diluted with 350 μl lysis buffer, then were added to pH 3–10 Linear strips 18 cm long. The strips were allowed to rehydrate for 12 h under mineral oil. The voltage was increased in the following steps: (1) voltage gradient 250 v for 30 min, (2) 500 v for 30 min, (3) 1,000 v for 1h, (4) 2,000 v for 1 h, (5) 4,000 v for 1 h, (6) 8,000 v for 3 h, and (7) a final phase of 8,000 v for 10 h. After electrophoresis was finished, The strips were equilibrated first by buffer A (1.5 M pH 8.8 Tris-HCL, 6 M urea, 20 % glycerol, 2 % SDS, 2 % DTT): and then by buffer B (1.5 M pH 8.8 Tris-HCL, 6 M urea, 20 % glycerol, 2 % SDS, 2.5 % acrylamide), both for 15 min before they were added to the top side of precast gel. Sodium dodecyl sulfate- polyacrylamide gel electrophoresis (SDS–PAGE) running was completed until the bromophenol blue front reached 1 cm from the bottom of the gel. The protein spots on the gels were stained with colloid CBB R-250. Gels were fixed for 2 h in 12 % trichloroacetic acid (TCA), submerged in colloid CBB R-250 solution (0.1 % CBB R-250, 40 % ethanol, 10 % ethanoic acid) for 16 h, and then washed with destaining solution (10 % ethanol, 10 % ethanoic acid). The stained gels were acquired using a gel image scanner (GS −800) and then processed by PDQUEST 7.30 2-DE-image analysis software.

### In-gel tryptic digest

The proteins spots were destained with 100 μl of 50 % NH_4_HCO_3_/5 0% ACN for 20 min twice, dehydrated with 100 μl ACN for 10 min, and digested with 3 μl of 12.5 mg/l trypsin digestion buffer at 4 °C. After 30 min of incubation, the gels were digested for over 12 h at 37 °C. The peptides were extracted with 60 μl of 50 % ACN+0.1 % TFA twice by incubation for 30 min. The extracted solutions of each spot were combined and then dried with nitrogen.

### MALDI-TOF-MS/MS and database search

For MALDI-TOF-MS, each digestion product was dissolved in 0.7 μl of matrix (5 g/l CHCA diluted in 50 % ACN + 0.1 % TFA), deposited onto a MALDI plate immediately and allowed to dry and crystallize at room temperature. MALDI-MS was performed. The instrument was operated in the positive ion reflection mode and batch mode acquisition control. The laser, 355 nm, was generated by Nd:YAG with accelerating voltage at 20 kV. Reflector spectra were obtained in the mass range of 700–3,500 D. The first six precursor ions with the highest intensity were selected for MS/MS analysis. All the values were calibrated and corrected by the values of two trypsin autolysis products of known amino acid sequences (m/z 842.510 and m/z 2, 211.105). The search engine was GPS–MASCOT in the “Sequence Query” model applying to both PMF and tandem MS. The parameters used were as follows: NCBI *Mus musculus*; searching mode as combined; 0.3 D mass tolerance for PMF and 0.4 D for tandem MS; monoisotopic, allowing one missed cleavage. The mass trypsin autolysis product and other stains were removed.

### Statistics

All data are expressed in mean±standard error (n=3). The significance of the difference between normal and inherited congenital cataract were tested with the Student *t*-test. If those spots with absolute differences about four folds greater, they were considered as upregulated. Vice versa. If those spots with absolute differences about one quater less, they were considered as downregulated. P values less than 0.05 are selected for further study.

## Results

### 2-DE electrophoresis

**Figure 1:**
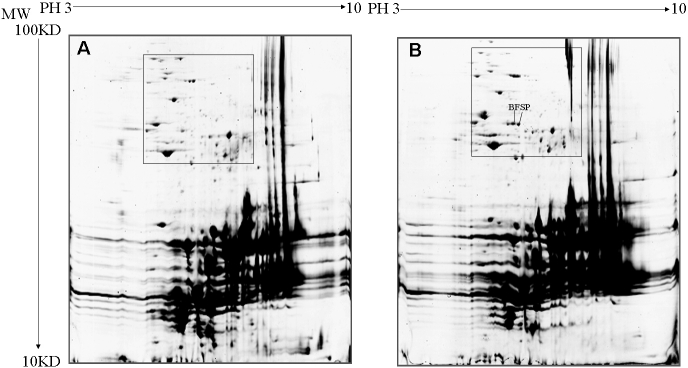
2-DE gels showing the proteome maps of total proteins from the lenses. 882 μg of samples were loaded. Pre-cast gels of 12 % were used for the second dimension, and were stained with colloid Coomassie brilliant blue (CBB) R-250. Low-abundance proteins were shown in the rectangle box. **A**: Cataract mice. BFSP/filensin spots were absent. **B**: Normal mice. BFSP/filensin spots were highly detectable in normal samples.

**Figure 2:**
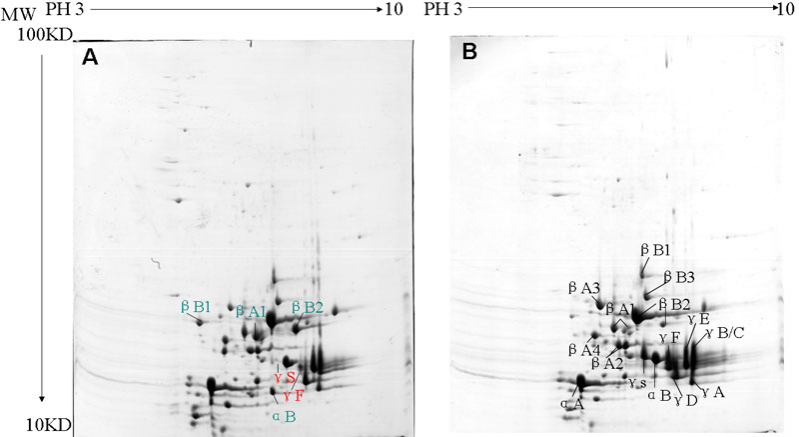
2-DE gel showing the differential proteins of lenses. 190 μg of samples were loaded. Pre-cast gels of 12.5 % were used for the second dimension, and were stained with colloid Coomassie brilliant blue (CBB) R-250. **A**: Six differential crystallins were identified from mutant mice. Red indicates absence (γS-crystallin) or reduction (γF-crystallin). Blue indicates increment (βA1-, βB1-, βB2-, and αB-crystallin). **B**: Sixteen crystallins were identified in normal mice, including αA~αB-, βA1~βA4-, βB1~βB3-, γA~γF-, and γS-crystallin.

When high dosage of samples, 882 μg, was loaded to the gel, 417±53 spots and 370±41 spots in cataract and normal lenses were observed, respectively. The difference was not statistically significant, however (p>0.05; Figure 1). When a low dose of sample, 190 μg, was loaded to the gel, 60±7 spots and 57±5 spots in cataract and normal lenses were observed, respectively. This difference also was not statistically significant (p>0.05; Figure 2).

### MALDI-TOF-MS/MS

**Figure 3:**
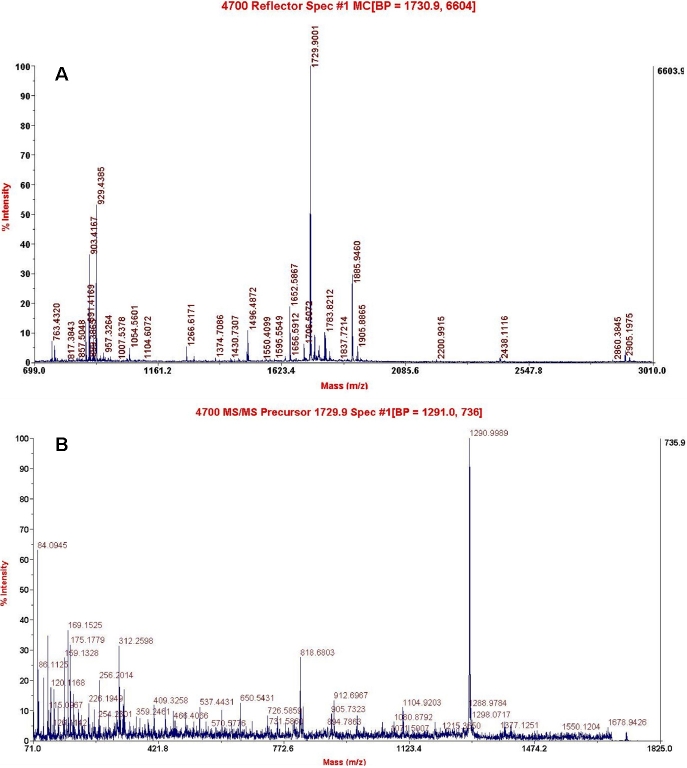
Identification of mouse γS-crystallin by mass spectrometry. **A**: MALDI-MS spectrum of the γS-crystallin spot digested with trypsin. **B**: Tandem MS spectrum of the ion with m/z 1729.9 from a tryptic peptide.

Seventeen crystallins were identified in normal lenses, such as αA- αB-, βA1- to βA4-, βB1- to βB3-, γA- to γF-, and γS-crystallin, and BFSP (Table 1). Besides βA1-, βB2-, γS-, γF-crystallin, and BFSP, another two differential crystallins, such as αB- and βB1-crystallin, were consistently identified in cataract lenses. In total, seven kinds of differential proteins were identified, including BFSP/filensin, γS-, γF-, βA1-, βB1-, βB2-, and αB-crystallin (Table 2). The spot representing BFSP/filensin was absent in the high dosage of cataract samples. However, it was highly detectable in normal samples (Figure 1). In the lower dosage of samples from mutant mice, γS-crystallin was not detected and γF-crystallin was downregulated while βA1-, βB1-, βB2-, and αB-crystallin were upregulated in the mutant cataract (Figure 2). The latter proteins had less molecular weight (MW) than normal, suggesting that they may have been lysed or truncated. The reduced isoelectric point (PI) of αB-crystallin suggested phosphorylation to the protein. The detailed results of γS-crystallin mass spectrometry are shown in Figure 3.

**Table 1:** Mass spectrometry results of 17 proteins in the normal lens.

**Protein name**	**Accession number**	**Relative MW**	**PI**	**Protein ion score**
αA	gi|387134	18525.3	5.86	107
αB	gi|14789702	20056.4	6.76	199
βA1	gi|20304089	25189.8	5.98	177
βA1	gi|20304089	25189.8	5.98	142
βA2	gi|10946978	22222.6	6.3	348
βA2	gi|10946978	22222.6	6.3	135
βA3	gi|109491386	25270.3	6.17	130
βA4	gi|10946672	22454.7	5.9	236
βB1	gi|12963789	27984.7	6.84	235
βB2	gi|6681035	23366.4	6.5	377
βB2	gi|6681035	23366.4	6.5	309
βB3	gi|10946674	24276.1	6.71	288
γA	gi|6724317	21148.8	7.54	195
γB/C	gi|2507570	21138.8	7.55	180
γD	gi|14861862	21103.6	6.99	210
γE	gi|27545356	21263.7	7.11	160
γF	gi|21746155	21234.8	6.78	318
γS	gi|6753532	20836.9	6.89	282
BFSP/filensin	gi|2754580	73583.8	5.81	352
BFSP/filensin	gi|2754580	73583.8	5.81	477

**Table 2:** Mass spectrometry results of 7 differential proteins.

**Protein name**	**Accession number**	**Relative MW**	**PI**	**Protein ion score**
γS	gi|6753532	20836.9	6.89	282
BFSP/filensin	gi|2754580	73583.8	5.81	477
γF	gi|21746155	21234.8	6.78	318
βB1	gi|2963789	27984.7	6.84	239
βA1	gi|20304089	25189.8	5.98	177
βB2	gi|6681035	23366.4	6.50	377
αB	gi|4789702	20056.4	6.76	124

## Discussion

In this study, seventeen major protein spots, including αA-, αB-, βA1- to βA4-, βB1- to βB4-, γA- to γF-, γS-crystallin, and BFSP/filensin, were identified in a 2-DE map of normal mouse lenses. In cataractous lenses, the spots representing γS-crystallin and BFSP/filensin could not be detected. γF-crystallin was downregulated while βA1-, βB1-, βB2-, and αB-crystallin were upregulated. Here, we discuss the crystallins that weren’t detected first and then those crystallins with abnormally high or low expression.

### γS-crystallin

Since this specific strain of mouse has a point mutation at *Crygs*, it is logical to consider γS-crystallin first. γS-crystallin is one member of the subfamily of γ-crystallins. Before 1989, it was considered as βS-crystallin [12]. With characteristics of both β- and γ-crystallins, γS-crystallin can serve as an indication of what a common ancestor to the β/γ-crystallins might have been like. Different from other γ-crystallins, whose genes usually are located on human chromosome 2, the gene for γS-crystallin is located on human chromosome 3 in humans and chromosome 16 in mice. Unlike other γ-crystallins, it possesses a short NH_2_-terminal arm with a blocked NH_2_-terminus like a β-crystallin. Otherwise, it closely resembles other γ-crystallins in its structure. Τhis crystallin exists as a monometric protein, like other γ-crystallins [12].

γS-crystallins are highly abundant in lenses and rarely found in corneas or retinas [10]. It is composed of 178 amino acids, with four beta foldings. It is highly conserved, suggesting a role in lens evolution. In the mice used in this study, there was a G489A mutation on their *Crygs* gene [11]. Position 163 is changed to a termination codon during translation. Theoretically, there should be a new protein with 162 amino acids. However, we did not find any spot that corresponded to this new protein, although the amount of normal γS-crystallins was greatly reduced. This absence of new proteins agreed with Jungblut’s study [13]. The mice used in that study had a mutation of the βB-crystallin gene. Theoretically, there should have been a new protein with 32 less amino acids, however; they did not find spots corresponding to this protein during 2-DE analysis.

γS-crystallin is rich in cysteines and is often modified by s-methylation. This prevents the exposed hydrogen sulfur from forming disufide-bonds, which makes the protein insolvable [14]. γS-crystallin also prohibits the aggregation of other γ-crystallins [15]. γS-crystallins act with α-crystallins to maintain the transparency of the lens [16]. When the production of γS-crystallins is reduced, it could block the process of nuclear extinction, which in turn may increase the proliferation and migration of the lens epithelial cells. The reduction and modification of γS-crystallins could also lead to age-related cataracts [17]. It has been reported that, when combined with carotenoid, γS-crystallins can absorb ultraviolet and blue lights in the retina. Similar to α-crystallins, γS-crystallin could also be a stress-induced protein, a subfamily of heat-shock proteins with small molecular weights [18]. α-Crystallins could also act as junior chaperones to other proteins to prevent aggregation caused by heating, ultraviolet light and chemicals. It also promotes the solubility of other proteins [19].

Previously, it has been reported that cataracts in Opj mice are related to the mutation of the *Crygs* gene. In contrast to the mice studied here, cataracts in Opj mice are autosomal dominant. Thus, not only homozygotes (Opj/Opj) show typical nuclear cataracts, as in the mice of this study, but heterozygotes also showed nuclear cataracts, although relatively less severe. When Opj mice age, the opacity of the lens becomes more pronounced. A single base change, from T to C, caused the replacement of Phe-9, a key hydrophobic residue in the core of the NH_2_-terminal domain, by serine [20].

### BFSP/filensin

The mass spectrometry analysis also revealed the absence of BFSP/filensin in the mice with inherited cataracts. This might have caused the disruption of the normal arrangement and connections among cells, as confirmed by the histological results from the electromicroscopy study. The intercellular spaces were much bigger and more irregular than those found in normal mice.

BFSP/filensin is one of the cytoskeletal proteins. It is only expressed in differentiated crystalline fibers. It provides the structural support for the cell’s movement and maintains the integrity of the cell shape. Normally functioning cytoskeletal proteins and their interactions to crystallins are essential to the development of the crystalline lens and the maintenance of its transparency. During conditions of stress, the small heat shock protein alphaB-crystallin may selectively target intermediate filaments for protection against unfolding [21]. The core components of BFSP include phakinin (BFSP-2, CP49) and filensin (BFSP-1, CP94, CP95, CP115). It has been reported that some cytoskeletal proteins are degraded during the earliest stages of cataract formation [22]. Jakobs et al. [23] reported the first case of inherited cataract caused by a mutation in cytoskeletal proteins in humans. In their study, a deletion mutation, DeltaE233, on the gene BFSP2, which encodes the lens-specific beaded filament protein, caused the structural changes to the protein and loss of the transparency of the lens.

### Other crystallins and their relationship to γS-crystallins

The various forms of the crystallins suggest that many different types of modification occur to the proteins after their translation stage. It is known that the posttranslational modifications and the following structural changes may reduce the solubility of the crystallins, ultimately leading to opacification of the lens [24]. As indicated by the positions of the spots of αB-crystallin, both the isoelectric point (PI) and MW of αB-crystallin were reduced in mutant mice compared to normal ones. The reduced PI suggested phosphorylation to the protein, and reduced molecular weight suggested truncation of the protein. The appearance of a new αB spot suggested both phosphorylation and truncation. Moreover, the fact that the positions of βB1-, βA1-, and βB2-crystallin were not overlapped with those in normal mice suggested that all three of these proteins were somehow lysed or truncated. In future studies, we plan to measure the sequence of those altered proteins in order to provide a better understanding of the changes to the crystallins. It is well known that, in mice with a mutation at *Crygs* gene, there is a reduced amount of γS-crystallin. However, in our study, we also found increased or decreased production of other crystallins. Those changes may be traced to the reduction of γS-crystallin since the spot mutation occurred at *Crygs*. The absence or reduction in the amount of γS-crystallin might disrupt the interaction between γS-crystallin and other crystallins, which is essential in maintaining transparency of the lens.

γS-crystallins were mainly located in the periphery cortex, which is transparent in nuclear cataracts. This finding suggests that the nuclear cataract is not directly caused by the γS-crystallin gene mutation. It is more likely to be the consequence of structural changes in other proteins following changes in γS-crystallins. The results from mass spectrometry analysis revealed the lysed βA1-, βB1-, βB2-, and αB-crystallin, which have lower solubility. Normal γS-crystallin not only prohibit the aggregation of γ-crystallins, but also prohibit the lysis of α- and β-crystallin. When the γS-crystallin is reduced or modified, the prohibitive effect is severely impaired. The absence of BFSP/filensin might also play an important role here.

The methods used herein have inherent limitations. Therefore, one has to be cautious in the interpretation of our data. For example, in 2-DE analysis, the isolation of proteins was solely based on the PI and MW. Although it is unlikely, sometimes within a protein spot there might be several different types of proteins with similar PI and MW. In our results, we did not find the *Crygs* mutated protein. This fact could be interpreted to mean that the mutated protein was missing. However; the possibility that the mutated protein was simply mixed with other protein spots could not be entirely ruled out. That is why, in this paper, we restrained ourselves from making certain definite claims that might be beyond the scope of the data attained through our methods. In future studies, we plan to apply other methods such as the identification of trace amount proteins and insoluble proteins to rule out those uncertainties and provide more definitive results.

In summary, we report that, in mutant *Crygs* mice, there were reduced γS-crystallins, absence of skeletonal protein (BFSP/filensin), downregulated γF-crystallin and increased lysis of βA1-, βB1-, βB2-, and αB-crystallin. However; the exact mechanism by which γS-crystallin caused the changes in other crystallins is still unknown. The alteration could happen during each stage of the protein synthetic cycle, such as transcription, translation, post-translational modification, and degradation. Reduced γS-crystallin, together with the absence of BFSP/filensin and downregulated γF-crystallin, suggested that γS-crystallin might be essential in the transcription and translation of BFSP/filensin and γF-crystallin. Whether this influence is at the DNA or mRNA level remains to be studied.
